# Sterile Inflammation and Cell Death Pathways in Liver Ischemia-Reperfusion Injury: A Review and Perspective

**DOI:** 10.2174/0118715303401342250514102731

**Published:** 2025-05-19

**Authors:** Weifan Huang, Wanting Meng, Jianan Zhao, Binbin Zhang

**Affiliations:** 1 Central Laboratory, Shuguang Hospital Affiliated to Shanghai University of Chinese Traditional Medicine, Shanghai, China;; 2 Academy of Integrated Medicine, Shanghai University of Traditional Chinese Medicine, Shanghai, China;; 3 Department of Rheumatology, Guanghua Hospital Affiliated to Shanghai University of Traditional Chinese Medicine, Shanghai, China;; 4 School of Clinical Medicine, Zhejiang University of Traditional Chinese Medicine, Hangzhou, China;; 5 Department of Hepatology, The Affiliated Hospital of Hangzhou Normal University, Hangzhou, China

**Keywords:** Ischemia-reperfusion injury, damage-associated molecular patterns, pharmacological strategies, therapeutic advances, next-generation therapies, systemic homeostasis

## Abstract

**Background:**

Hepatic Ischemia-Reperfusion Injury (IRI) is a critical complication in liver transplantation and resection, driven by oxidative stress and sterile inflammation mediated by damage-associated molecular patterns (DAMPs). Current therapeutic challenges arise from interconnected cell death pathways and redundant inflammatory mechanisms.

**Objective:**

This review synthesizes mechanistic insights into DAMP signaling and regulated cell death modalities in IRI, aiming to identify translational gaps and propose precision-targeted therapies.

**Methods:**

A literature search in PubMed using keywords “IRI,” “DAMPs,” and cell death modes was conducted without date restrictions. Peer-reviewed studies on human/animal models were included, with qualitative synthesis of DAMP-cell death interactions.

**Results:**

During ischemia, mitochondrial dysfunction releases HMGB1, ATP, and mtDNA, activating Kupffer cell TLR4/RAGE and cGAS-STING pathways, triggering NLRP3 inflammasome- driven cytokine storms. Reperfusion amplifies ROS bursts, lipid peroxidation, and iron overload, creating a self-sustaining cycle of damage. Cell death modalities exhibit spatiotemporal specificity: hepatocyte ferroptosis dominates early injury, while macrophage pyroptosis and necroptosis predominate in steatotic livers during late phases. HMGB1 lactylation and mtDNA-cGAS signaling emerge as key regulators. Machine perfusion (*e.g.*, hypothermic oxygenated perfusion) reduces biliary complications *via* mitochondrial resuscitation, outperforming conventional drug-based therapies.

**Conclusion:**

Current single-pathway targeting shows limited efficacy due to IRI’s complexity. Future strategies should integrate temporal targeting (ferroptosis inhibitors pre-reperfusion; pyroptosis blockers post-reperfusion), DAMP-neutralizing agents (anti-HMGB1 antibodies), and precision preservation combining multi-omics biomarkers with *ex vivo* pharmacological preconditioning. Addressing metabolic vulnerabilities in fatty livers and refining cell death-specific interventions are critical for bridging translational gaps.

## 1 INTRODUCTION

The liver, the largest internal organ in vertebrate organisms, orchestrates an intricate network of physiological processes essential for systemic homeostasis. Beyond its canonical roles in protein synthesis and glucose-lipid metabolism, this multifunctional organ serves as the body's principal detoxification center. Hepatic detoxification mechanisms, primarily mediated through phase I (cytochrome P450 enzymes) and phase II (conjugation enzymes) biotransformation pathways, enzymatically convert lipophilic toxins into water-soluble metabolites for subsequent biliary or urinary excretion [1]. Concurrently, the liver functions as an immunological sentinel, where tissue-resident macrophages (Kupffer cells) coordinate with recruited bone marrow-derived monocytes to eliminate bloodborne pathogens and cellular debris [2]. This functional complexity renders the liver uniquely vulnerable to ischemia-reperfusion injury (IRI), a biphasic pathological cascade initiated by oxygen deprivation followed by inflammatory exacerbation upon blood flow restoration [3]. Clinically prevalent in transplantation (60-70% of cadaveric liver transplants), extended hepatectomy (>50% parenchymal resection), and hemorrhagic shock management, IRI manifests as mitochondrial oxidative stress, sinusoidal endothelial cell necrosis, and subsequent activation of pro-apoptotic pathways [4]. According to ELTR registry data, the resultant parenchymal damage frequently progresses to postoperative hepatic insufficiency, accounting for 38% of early graft failures in transplant recipients [4].

Sterile inflammation represents a pathogen-independent immune activation triggered by endogenous danger signals during tissue stress or injury-induced responses [5, 6]. This self-perpetuating process originates from necrotic cell death or mechanical disruption, liberating damage-associated molecular patterns (DAMPs) that function as molecular signatures of compromised cellular integrity [7]. Released DAMPs including HMGB1, ATP, and mitochondrial DNA - engage pattern recognition receptors (PRRs) on sentinel immune cells, notably macrophages and dendritic cells, through TLR4 and NLRP3 inflammasome activation pathways [7]. Such interactions initiate cascades of pro-inflammatory cytokine production (IL-1β, TNF-α) and chemokine release, orchestrating both local and systemic inflammatory amplification while coordinating tissue repair mechanisms. Distinct from infection-driven responses, sterile inflammation operates through PRR-mediated detection of intracellular components leaked during non-programmed cell death [8]. Its pathophysiological spectrum encompasses ischemia-reperfusion injury cascades, metabolic dysregulation states (*e.g.*, NASH in obesity, diabetic nephropathy), autoimmune activation, and tumor microenvironment remodeling. Clinical manifestations range from transient post-traumatic swelling to chronic inflammatory progression in cardiometabolic diseases and carcinogenesis.

The liver possesses unique immunological and metabolic features that contribute to its heightened susceptibility to IRI. One key characteristic is its high density of Kupffer cells, which are resident macrophages in the liver and play a central role in the initiation of sterile inflammation [9]. Kupffer cells are strategically positioned to rapidly detect and respond to DAMPs released from injured tissues [10]. Moreover, the liver's exposure to DAMPs is exacerbated by the portal venous system, which directly links the liver to the gastrointestinal tract, a major source of microbial products and DAMPs. This exposure, combined with the liver's immunological environment, creates a unique setting where the inflammatory response is both amplified and prolonged during IRI. Unlike other organs, such as the heart or kidneys, the liver’s ability to rapidly clear DAMPs *via* the biliary epithelium further distinguishes its inflammatory response [11]. The interaction between Kupffer cells, sinusoidal endothelial cells, and the hepatic microvasculature enhances the liver’s vulnerability to IRI, making it more prone to sustained inflammation and cellular damage after ischemic insult [12, 13].

Sterile inflammation constitutes the central pathogenic axis in hepatic ischemia-reperfusion injury (IRI), mediated through coordinated DAMP signaling and redox imbalance. Hepatocyte necrosis during the ischemic phase releases nuclear HMGB1 and mitochondrial DNA fragments that engage TLR9 and RAGE receptors on Kupffer cells, priming the NLRP3 inflammasome machinery [7]. Subsequent reperfusion accelerates reactive oxygen species (ROS) generation *via* xanthine oxidase and compromised mitochondrial electron transport chains, establishing oxidative stress thresholds that activate NF-κB and MAPK inflammatory pathways [14]. This dual-phase mechanism generates cytokine storms (IL-6, IL-18) and chemotactic gradients (CXCL1, CCL2) that recruit neutrophils and Ly6C^+^ monocytes, perpetuating parenchymal destruction through NETosis and ferroptosis cascades. Therapeutic targeting of DAMP clearance mechanisms or mitochondrial ROS quenching has demonstrated efficacy in preclinical models, highlighting translational potential through inflammasome modulation and antioxidant pathway potentiation.

Current therapeutic strategies targeting hepatic ischemia-reperfusion injury focus on three mechanistic pillars: redox homeostasis restoration (*e.g.*, N-acetylcysteine, superoxide dismutase mimetics), inflammation modulation (IL-1 receptor antagonists, NLRP3 inhibitors), and mitochondrial stabilization (cyclosporine A, mdivi-1) [15-19]. Clinical translation of pharmacological preconditioning approaches demonstrates variable efficacy: while prostaglandin-based vasodilation (PGE1/PGI2) showed hepatoprotective effects in transplant models by enhancing sinusoidal perfusion [20, 21], while immunomodulatory strategies like post-transplant thymoglobulin administration improved early graft function in pilot studies [22]. Contrastingly, a recent multicenter trial testing combined vasodilators, anticoagulants, and antioxidants showed no significant reduction in IRI biomarkers [23]. The lack of consensus on therapeutic protocols underscores the urgent need for large-scale clinical trials validating preclinical findings and addressing interspecies translational gaps. Prioritizing human-relevant models and stratified patient cohorts will accelerate the development of novel, mechanism-driven therapies for IRI.

In this article, we will summarize recent advances in the understanding of DAMPs and related cell death in liver ischemia-reperfusion injury. We will also discuss cutting-edge progress in this field, with the aim of providing valuable insights for clinical treatment strategies.

## 2 STUDY METHODOLOGY

Search Strategy: A systematic literature search was conducted in PubMed using keywords including “ischemia-reperfusion injury (IRI),” “damage-associated molecular patterns (DAMPs),” DAMP-related molecules, and terms for cell death modalities (*e.g.*, ferroptosis, pyroptosis, necroptosis). No date restrictions were applied during the initial screening to ensure comprehensive coverage of relevant studies.

Study Selection: Two independent reviewers performed manual screening and full-text evaluation. All peer-reviewed articles directly addressing DAMPs and cell death mechanisms in liver IRI were included, regardless of publication type. Studies unrelated to hepatic IRI pathophysiology or published in non-English languages were excluded through consensus decision-making.

Data Extraction and Synthesis: As this review does not constitute a systematic review or meta-analysis, no formal quantitative data extraction or statistical reanalysis was performed. Key findings from included studies were synthesized narratively, with citations based on the original studies' findings and contextual relevance. Mechanistic frameworks and therapeutic implications were derived through critical analysis of experimental evidence and clinical observations across referenced literature.

## 3 THE PATHOGENESIS OF LIVER ISCHEMIA- REPERFUSION

The pathophysiology of hepatic Ischemia-Reperfusion Injury (IRI) evolves through two distinct yet interconnected phases: ischemic priming and reperfusion exacerbation [4]. During ischemia, oxygen/nutrient deprivation induces mitochondrial dysfunction, depleting ATP stores below critical thresholds required for membrane ion pump activity [24]. This metabolic collapse drives intracellular calcium overload, activating calpain proteases and mitochondrial permeability transition pores that initiate programmed apoptosis or accidental necrosis [24].

Reperfusion paradoxically amplifies tissue damage through two synergistic mechanisms. First, oxygen resurgence fuels xanthine oxidase-mediated superoxide production, generating cytotoxic reactive oxygen species (ROS) that induce lipid peroxidation, protein carbonylation, and mitochondrial DNA damage [14]. Second, ischemic preconditioning activates Kupffer cells to release TNF-α and IL-8, priming neutrophil recruitment through CXCL1/CXCR2 chemotaxis [25]. Post-reperfusion, infiltrating leukocytes propagate a cytokine storm (TNF-α, IL-1β) *via* TLR4/MyD88 signaling while releasing secondary ROS through NADPH oxidase activation [5]. This self-reinforcing cycle disrupts redox homeostasis, exacerbating endoplasmic reticulum stress and ferroptosis through GPX4 inactivation. The interplay between oxidative burst and sterile inflammation creates a pathological feedback loop, ultimately determining parenchymal survival *versus* failure.

## 4 DAMPS IN LIVER ISCHEMIA REPERFUSION INJURY

**Fig. (1):**
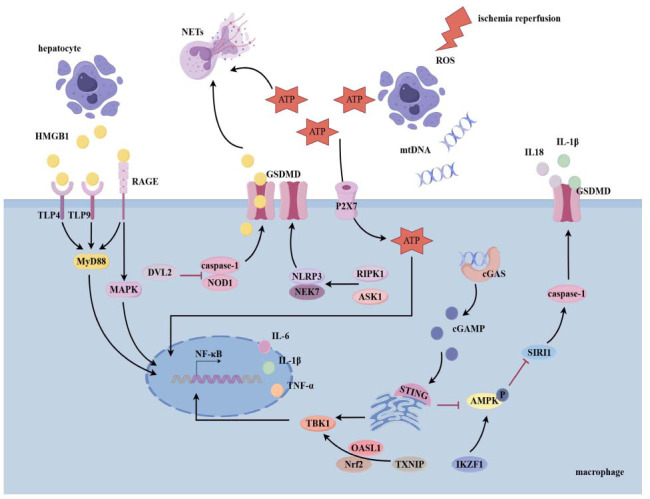
DAMPs in liver ischemia reperfusion injury.

Damage-associated molecular patterns (DAMPs) are endogenous danger signals released during cellular stress or injury, functioning as key mediators of sterile inflammation in IRI [13]. These intracellular components-including nuclear DNA, mitochondrial proteins, and chromatin-associated molecules-become immunogenic upon extracellular release, activating pattern recognition receptors on Kupffer cells and recruited leukocytes [7]. Hepatocyte-derived DAMPs are particularly critical in IRI progression, driving both innate immune activation and parenchymal damage amplification through autocrine and paracrine signaling loops (Fig. **1**).

### 4.1 High Mobility Group Box 1 (HMGB1)

HMGB1, a 215-amino acid nuclear protein containing two DNA-binding domains (A-box and B-box) and an acidic C-terminal tail, undergoes compartmentalization-dependent functional switching [26]. While nuclear HMGB1 regulates chromatin stability, cytosolic translocation during ischemic stress precedes its extracellular release as a prototypic DAMP [27]. In hepatic IRI, ischemia-induced acetylation promotes HMGB1 cytoplasmic accumulation, with serum levels escalating progressively during reperfusion [28]. Liberated HMGB1 engages TLR4 and RAGE on sinusoidal endothelial cells, activating MyD88-dependent NF-κB signaling that triggers neutrophil extracellular trap (NET) formation and macrophage pyroptosis [29, 30].

Mechanistic studies reveal HMGB1’s dual role in ferroptosis regulation: disulfide HMGB1 upregulates ferroportin *via* TLR4/RAGE to potentiate iron overload [31], while macrophage-specific RIPK1/ASK1 deletion suppresses NLRP3 inflammasome-mediated HMGB1 release, mitigating lipid peroxidation [32, 33]. Therapeutic targeting of HMGB1 dynamics shows clinical relevance—hepatocyte-specific HSPA12A overexpression attenuates IRI by inhibiting glycolysis-driven HMGB1 lactylation and subsequent secretion [34]. It does so by suppressing glycolysis-mediated HMGB1 lactylation and the subsequent secretion of HMGB1 by hepatocytes [34]. Paradoxically, neutrophil-derived HMGB1 exhibits context-dependent immunomodulation: HMGB1-deficient neutrophils demonstrate hyperactivated ROS production and exacerbated inflammation during early transplantation injury [35]. Pharmacological blockade of HMGB1-TLR4 interactions (*e.g.*, eritoran) reduces Kupffer cell activation and represents a promising therapeutic strategy [29].

### 4.2 Adenosine Triphosphate (ATP)

ATP serves dual roles as a cellular energy currency and a key DAMP in hepatic ischemia-reperfusion injury [36]. During ischemic phases, metabolic crisis (hypoxia, acidosis, ATP depletion) forces hepatocytes to rely on accelerated glycogenolysis, exacerbating mitochondrial ROS generation and calcium overload through SERCA pump dysfunction [37]. Upon the release of ATP from injured hepatocytes, GATA6-positive peritoneal macrophages are attracted to the site of injury *via* the trans-capsular route rather than through blood vessels to clear necrotic cells and contribute to the vascular remodeling of the damaged tissue [38]. Under cellular injury and inflammatory stress, extracellular ATP functions as a DAMP, inducing potassium ion efflux and activating NLRP3 inflammasomes by binding to P2X7 receptors on immune cells [39]. Simultaneously, ATP released from necrotic hepatocytes can activate immune cells, such as Kupffer cells, thereby amplifying the inflammatory response [40]. On the flip side, intracytoplasmic ATP produced by CKB is required for NLRP3 activation [41]. Upon hepatic blood reperfusion, circulating DAMPs, such as ATP and HMGB1, activate neutrophils and recruit them to the site of injury, thereby promoting the formation of neutrophil extracellular traps [30, 40]. Additionally, HMGB1 and ATP may synergistically activate NLRP3 inflammasomes, with HMGB1 initiating the process and ATP inducing potassium efflux, thereby triggering inflammation [42, 43].

### 4.3 mtDNA

Hepatic ischemia-reperfusion injury induces cellular stress that compromises nuclear and mitochondrial membrane integrity, leading to extracellular DNA release. This extracellular DNA is detected by pattern recognition receptors, particularly cyclic GMP-AMP synthase (cGAS), which initiates innate immune signaling cascades [44, 45]. Upon binding cytoplasmic DNA, cGAS undergoes structural activation to catalyze cyclic GMP-AMP (cGAMP) synthesis, a secondary messenger critical for immune activation [46]. The resultant cGAMP engages the endoplasmic reticulum-resident stimulator of interferon genes (STING) through its cytoplasmic domain, inducing oligomerization and subsequent activation of this transmembrane adaptor protein [47, 48]. The activated STING pathway coordinates hepatic inflammation through two principal downstream effectors: NF-κB drives pro-inflammatory cytokine production, while IRF3 initiates type I interferon responses, collectively exacerbating tissue apoptosis and inflammatory damage [46].

Emerging evidence underscores the pivotal involvement of macrophage-derived cGAS-STING signaling in HIRI pathogenesis. Although hepatocytes exhibit minimal STING expression under ischemic conditions, hepatic macrophages demonstrate substantial STING upregulation during reperfusion injury [49]. Myeloid-specific STING activation exacerbates metabolic dysregulation through HIF1-α accumulation and AMPK signaling suppression, establishing a pro-inflammatory microenvironment [49]. Mechanistic studies reveal that macrophage thioredoxin-interacting protein (TXNIP) coordinates STING-TBK1 activation *via* NRF2-OASL1 pathway modulation, directly linking oxidative stress responses to innate immune activation in ischemia-challenged livers [50]. Therapeutic interventions targeting mitochondrial quality control demonstrate that Mixed lineage kinase domain-like protein (MLKL) deficiency enhances PINK1-mediated mitophagy, thereby reducing hepatocyte DNA leakage and subsequent macrophage cGAS-STING activation [51]. Furthermore, SIRT3 inhibition amplifies hepatic injury by facilitating p65-mediated cGAS transcriptional upregulation, creating a pathogenic feedback loop that sustains STING pathway activation [52].

### 4.4 Other Potential New Types of DAMPs

In addition to the common DAMPs mentioned above, recent studies have highlighted the roles of various other DAMPs in liver diseases. Extracellular matrix proteins, when released during liver injury, can activate immune cells *via* TLR2 and TLR4, thereby promoting inflammatory responses in the liver [53, 54]. Heat shock proteins, an important class of molecular chaperones, are released under conditions of cellular stress. Through binding to TLR2 and TLR4, they induce immune system activation, which in turn triggers aseptic inflammation [55, 56].

With the increasing prevalence of metabolic diseases in recent years, excessive fatty acid accumulation due to metabolic abnormalities leads to the rupture of fat droplets and the release of DAMPs, which, in turn, promote inflammation through TLR4-mediated pathways [57, 58]. Members of the S100 protein family, particularly S100A8 and S100A9, are crucial immunomodulatory factors that play key roles in liver injury and inflammation. The expression of S100A8/A9 has been found to be significantly elevated in both acute liver injury and chronic inflammation [59-61]. These proteins activate macrophages and other immune cells *via* TLR4, exacerbating inflammatory responses and promoting the development of hepatic fibrosis [62].

## 5 STERILE INFLAMMATION-INDUCED CELL DEATH IN LIVER ISCHEMIA-REPERFUSION INJURY

**Fig. (2):**
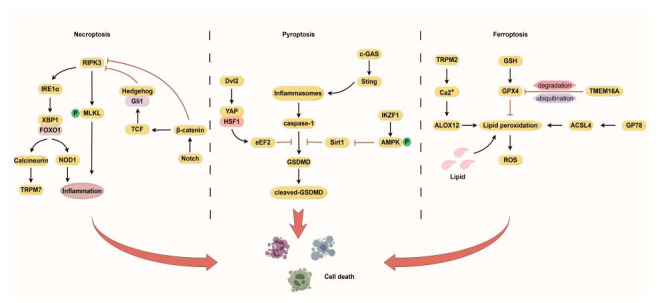
Sterile inflammation-induced cell death in liver ischemia reperfusion injury.

The pathophysiology of hepatic ischemia-reperfusion injury (IRI) involves a spectrum of regulated cell death modalities that synergistically propagate tissue damage through DAMP release. As outlined earlier, DAMP generation in IRI predominantly originates from cellular demise triggered by ischemia-reperfusion (IR) stress, with apoptosis, necrosis, necroptosis, pyroptosis, and ferroptosis constituting the principal lethal pathways observed in clinical and experimental settings [63]. Notably, these cell death mechanisms frequently exhibit spatiotemporal overlap and molecular crosstalk, generating interconnected lethal networks that amplify parenchymal injury. While homeostatic apoptosis facilitates immunologically silent clearance through phagocyte-mediated efferocytosis, dysregulated apoptotic signaling-particularly when efferocytosis capacity is overwhelmed by excessive apoptotic cells-progresses to secondary necrosis with compromised membrane integrity, culminating in DAMP leakage and initiation of sterile inflammatory cascades [64-66]. Given the well-characterized role of apoptosis in IRI, this section will focus on emerging evidence implicating non-apoptotic cell death pathways as central executors of IR-induced hepatocellular damage (Fig. **2**).

### 5.1 Necroptosis

Necroptosis represents a caspase-independent programmed necrosis pathway governed by the RIPK1-RIPK3-MLKL signaling axis, distinct from classical apoptotic cascades [67]. Intercellular crosstalk between hepatic macrophages and parenchymal cells critically regulates necroptotic signaling amplification during ischemia-reperfusion injury. Mechanistically, macrophage-derived RIPK3 potentiates NOD1-dependent inflammation through dual activation of the unfolded protein response effector IRE1α-XBP1 and FOXO1 transcriptional signaling [68]. Complementary studies demonstrate that macrophage NOTCH1 orchestrates β-catenin-dependent coordination of TAK1-mediated innate immunity and RIPK3-driven hepatocyte necroptosis [69]. This regulatory paradigm extends to bone marrow-derived immune cells, where the FOXO1-β-catenin-Hedgehog/Gli1 signaling axis modulates stress-responsive hepatic inflammation and necroptosis thresholds [70]. The pathophysiological relevance of necroptosis is particularly pronounced in steatotic liver transplantation models. Lipid-overloaded hepatocytes exhibit enhanced susceptibility to MLKL-mediated membrane permeabilization [71], a phenotype attenuated by the necroptosis inhibitor VX765 through suppression of immune cell recruitment and subsequent HFD-aggravated IR injury [72]. Intriguingly, temporal specificity governs the therapeutic targeting of this pathway, with RIPK3 inhibition demonstrating selective efficacy against late-phase injury progression rather than early ischemic events [71].

### 5.2 Pyroptosis

Pyroptosis, a lytic programmed cell death mechanism prevalent in myeloid cells, executes inflammatory signaling through NLRP3/AIM2 inflammasome activation and caspase-1/11-dependent cleavage of gasdermin D (GSDMD) [73]. These cytosolic sensors amplify tissue damage when hyperactivated, driving GSDMD oligomerization to form plasma membrane pores that release damage-associated molecular patterns (DAMPs), including high-mobility group box 1 (HMGB1), into the extracellular milieu [74]. While hepatocytes exhibit GSDMD-independent responses to ischemia-reperfusion stress [75], myeloid-specific GSDMD pore formation emerges as a central driver of sterile inflammation. Hepatic macrophages demonstrate coordinated regulation of pyroptotic pathways, evidenced by ischemia-induced upregulation of Ikaros (IKZF1), which suppresses SIRT1 *via* AMPK signaling to potentiate inflammasome activation and pyroptosis [76]. Regulatory crosstalk between innate immune receptors further modulates pyroptotic thresholds. NOD1 activation in Kupffer cells initiates caspase-1/GSDMD cleavage and IL-1β maturation, amplifying inflammatory cascades [77]. Conversely, macrophage dishevelled-2 (Dvl2) exerts protective effects by facilitating YAP-HSF1 complex formation, which induces heat shock factor 1 (HSF1)-dependent eEF2 transcription to suppress NOD1-mediated GSDMD activation [32]. Mitochondrial dysfunction exacerbates this axis, as hepatocyte-derived mitochondrial DNA (mtDNA) activates macrophage cGAS-STING signaling to upregulate NLRP3 expression, thereby licensing GSDMD-dependent pyroptosis and perpetuating parenchymal injury [78-80]. Therapeutic targeting of this pathway demonstrates context-dependent efficacy. In steatotic livers predisposed to severe IRI, pharmacological inhibition of lytic cell death significantly attenuates tissue damage [72], underscoring the clinical relevance of pyroptosis in metabolically compromised transplants. Notably, while myeloid GSDMD activation is firmly established as pathogenic, hepatocyte-specific roles of GSDMD remain enigmatic, warranting investigation into cell-type-specific gasdermin signaling during ischemic stress.

### 5.3 Ferroptosis

Ferroptosis, an iron-dependent programmed cell death mechanism distinct from apoptosis or necrosis, is characterized by glutathione (GSH) depletion, glutathione peroxidase 4 (GPX4) inactivation, and lethal lipid peroxidation. The liver’s inherent susceptibility to oxidative stress and iron accumulation renders it particularly vulnerable to ferroptotic damage during ischemia-reperfusion injury (IRI). Mechanistically, dysregulated iron metabolism disrupts redox homeostasis, driving reactive oxygen species (ROS) accumulation and peroxidation of polyunsaturated fatty acid (PUFA)-containing phospholipids. Core regulatory axes include the GPX4-mediated antioxidant defense system, SLC7A11-xCT cystine/glutamate antiporter activity, and transcriptional regulators such as NRF2, p53, and ACSL4, which collectively govern cellular ferroptosis thresholds [81]. Emerging studies delineate molecular drivers amplifying ferroptosis in IRI. Transmembrane protein TMEM16A promotes GPX4 ubiquitination and degradation [82], while E3 ubiquitin ligase GP78 enhances ACSL4 stability to potentiate lipid peroxidation [83]. Oxidative stress further exacerbates ferroptosis *via* TRPM2-mediated calcium influx, which upregulates arachidonate 12-lipoxygenase (ALOX12) to accelerate membrane lipid breakdown [9, 84].

The pharmacological targeting of ferroptosis demonstrates therapeutic promise. Small-molecule inhibitors like AS-252424 block ACSL4 enzymatic activity to suppress lipid peroxidation [85], while GPR56 agonist 17α-hydroxypregnenolone enhances CD36 degradation, reducing PUFA substrate availability for peroxidation [86]. Metabolic reprogramming strategies, including PTEN inhibition or mTOR activation, elevate NADPH *via* malic enzyme 1 (Me1) to restore redox balance [87]. Similarly, AHR antagonist CH223191 attenuates ferroptosis by suppressing the pro-oxidant HO1/COX2 axis [88].

The interplay between hepatic steatosis and ferroptosis exacerbates IRI severity. Lipid-laden hepatocytes exhibit heightened susceptibility to iron-mediated peroxidative damage, with ferroptosis inhibitors (*e.g.*, ferrostatin-1) significantly mitigating injury in fatty liver models [72]. This metabolic vulnerability highlights ferroptosis as a critical therapeutic node in steatotic liver transplantation. Despite advances, key knowledge gaps persist regarding cell-type-specific ferroptosis regulation (*e.g.*, hepatocytes *vs*. Kupffer cells) and temporal activation patterns during ischemic phases. Future investigations should prioritize multi-targeted strategies addressing iron metabolism, lipid homeostasis, and antioxidant defense systems to combat this complex cell death modality.

## 6 CLINICAL TRIALS RELATED TO LIRI

Clinical trials targeting liver ischemia-reperfusion injury (IRI) have evolved along two strategic axes: pharmacological modulation of injury pathways and technological optimization of graft preservation. These parallel approaches reflect the growing recognition of IRI's multifactorial pathogenesis, requiring combinatorial strategies to improve transplant outcomes.

### 6.1 Pharmacological Interventions

Antioxidant therapies exemplify the challenges of single-pathway targeting. While N-acetylcysteine (NAC) demonstrated preclinical efficacy in neutralizing reactive oxygen species, a randomized controlled trial (Meurisse et al.) revealed limited clinical benefit when administered post-transplantation, underscoring the temporal constraints of oxidative damage mitigation [23]. On the other hand, the failure of NAC may be attributed to “time constraints”, and the pharmacokinetic limitations were not thoroughly explored. Anti-inflammatory strategies show greater promise, with prostaglandin E1 administration during transplantation reducing IL-6-mediated graft dysfunction through Kupffer cell modulation [89]. TNF-α inhibition *via* infliximab, though mechanistically sound in preclinical models, exhibited suboptimal efficacy in a combined drug regimen targeting cold-preserved grafts, highlighting critical limitations in therapeutic timing and pathway redundancy [23, 90-92]. These outcomes emphasize the necessity for stage-specific interventions coordinated with ischemia-reperfusion phases.

### 6.2 Machine Perfusion Advancements

Dynamic preservation technologies address IRI through the physiological maintenance of donor's livers. Normothermic machine perfusion (NMP) maintains metabolic activity *via* oxygenated blood perfusion at 37°C, enabling functional viability assessment while reducing mitochondrial-derived oxidative stress. Multicenter trials demonstrate NMP's capacity to decrease early allograft dysfunction (EAD) and improve marginal graft utilization compared to static cold storage [93, 94]. Complementing this approach, hypothermic oxygenated perfusion (HOPE) targets mitochondrial resuscitation through controlled reoxygenation at 4-12°C. A landmark RCT revealed HOPE's superior protection against biliary complications, reducing non-anastomotic strictures through the inhibition of neutrophil extracellular trap formation [95]. Both modalities synergistically mitigate IRI *via* distinct mechanisms: NMP sustains physiological homeostasis, while HOPE prevents cold-induced metabolic collapse.

### 6.3 Translational Challenges and Future Directions

Current clinical limitations stem from three key factors: 1) Heterogeneity in donor-recipient metabolic profiles, 2) Temporal mismatch between intervention and injury phase, and 3) Redundant inflammatory pathway activation. The failure of combined drug regimens [72] reinforces the need for biomarker-guided personalized approaches. Emerging strategies integrating machine perfusion with *ex vivo* pharmacological preconditioning-such as HOPE-mediated delivery of siRNA targeting complement components-represent a paradigm shift in graft optimization. Large-scale multicenter trials incorporating multi-omics profiling (*e.g.*, mitochondrial respiratory signatures, and lipid peroxidation markers) are critical for identifying responsive patient subgroups and refining therapeutic sequencing [23].

In the treatment of IRI, both mechanical perfusion techniques and pharmacological strategies offer distinct potential, but each faces unique challenges. Mechanical perfusion techniques, particularly in liver transplantation, can effectively reduce liver damage caused by IRI, as well as improve donor liver function and survival by maintaining blood flow to the organ during ischemia. This approach helps minimize cellular damage during ischemia by providing a continuous supply of oxygen and nutrients, thereby facilitating the subsequent reperfusion recovery process. However, the effectiveness of mechanical perfusion depends on the complexity of the equipment and the technical proficiency required for its operation. Additionally, it is costly and may not fully eliminate the immune response associated with IRI.

In contrast, pharmacological strategies aim to modulate cellular and molecular mechanisms to attenuate inflammation and oxidative stress induced by IRI. These strategies include the use of antioxidants, anti-inflammatory agents, and other pharmacological interventions to regulate liver cellular responses and promote repair processes. Pharmacological approaches are highly flexible in their application, relatively easy to implement, and more cost-effective. However, their efficacy is often constrained by factors such as the therapeutic window, dose control, and the specificity and potency of the drugs used.

Overall, mechanical perfusion techniques provide immediate physiological support and reduce direct injury caused by hepatic ischemia, while pharmacological strategies focus on mitigating inflammation and cellular damage at the molecular level. When used together, these approaches can complement one another to improve clinical outcomes.

## CONCLUSION 

Hepatic ischemia-reperfusion injury (IRI), a common complication in liver transplantation, hepatic surgery, and management of liver diseases, embodies a paradoxical interplay between ischemic insult and reperfusion-triggered exacerbation of tissue damage. Central to this pathology is the release of damage-associated molecular patterns (DAMPs)-including nuclear DNA, mitochondrial RNA, HMGB1, and heat shock proteins-from stressed or dying hepatocytes. These endogenous danger signals activate pattern recognition receptors on immune cells, initiating cytokine storms that amplify sterile inflammation and parenchymal injury. Emerging therapeutic strategies targeting DAMP containment, such as anti-NINJ1 antibodies that inhibit plasma membrane rupture [96, 97], demonstrate the potential of membrane stabilization to disrupt this vicious cycle.

The hepatocyte death continuum in IRI encompasses apoptosis, necrosis, pyroptosis, and ferroptosis, each contributing uniquely to DAMP release. Necrotic membrane rupture facilitates passive leakage of intracellular ATP and histones, while pyroptosis employs NLRP3 inflammasome- mediated GSDMD pore formation for orchestrated IL-1β secretion. Ferroptosis, driven by iron-dependent lipid peroxidation, generates oxidative breakdown products that synergize with mitochondrial DAMPs to fuel neutrophil recruitment. Notably, ferroptosis regulators (*e.g.*, GPX4, ACSL4) and pyroptosis effectors (*e.g.*, caspase-1, GSDMD) exhibit cell- type-specific dominance, with hepatocytes favoring ferroptotic pathways and macrophages driving pyroptotic inflammation.

Current therapeutic limitations stem from monotherapeutic approaches targeting single pathways, which are insufficient to address the redundancy of DAMP release mechanisms. Promising strategies include combinatorial inhibition of GSDMD pores (*e.g.*, VX765) and ferroptotic lipid peroxidation (*e.g.*, ferrostatin-1), alongside DAMP-neutralizing interventions such as recombinant HMGB1 antibodies or DNase-I-mediated mtDNA clearance. Metabolic preconditioning *via* the pharmacological activation of SIRT3 or AMPK may further enhance cellular stress resilience. Future research must prioritize spatiotemporal mapping of dominant cell death modalities across ischemic (ferroptosis-predominant) and reperfusion (pyroptosis/necroptosis-driven) phases, organelle-centric targeting of mitochondrial permeability transition pores or ER stress sensors (*e.g.*, IRE1α), and machine learning-driven biomarker panels integrating cfDNA, extracellular HMGB1, and lipid peroxides for personalized therapeutic timing.

Although numerous preclinical studies in recent years have successfully identified a range of potential therapeutic strategies (*e.g.*, drugs, cellular therapies, *etc*.), the translation from preclinical models to clinical trials remains fraught with challenges.

First, the use of animal models in IRI presents significant obstacles when translating findings to clinical trials. Preclinical studies often employ mouse, rat, or pig models to simulate IRI, but these animals differ from humans in terms of physiology, metabolism, and immune responses [98]. For example, some therapeutic strategies that demonstrate significant effects in animal models fail to replicate the same results in clinical trials. This discrepancy may be attributed to physiological differences, as small animal models often do not fully replicate the complex structure and function of the human liver, especially in chronic disease states or advanced age. Second, the dose and timing of therapeutic interventions are crucial considerations. Many preclinical studies focus on a single time point or dose range for a drug or intervention. However, in clinical trials, individual patient variability, disease states, and treatment windows complicate the effective application of these standardized regimens in real-world settings. Particularly, the inconsistency of a drug's dose-response curve between animal models and humans is a common issue, often resulting in preclinical findings failing to translate successfully into clinical outcomes. Moreover, the diversity of preclinical models adds another layer of complexity. Although various animal models of hepatic IRI exist (*e.g.*, total hepatic ischemia, localized ischemia), these models may not adequately represent all clinical scenarios. For instance, IRI in liver transplant patients may differ from IRI resulting from surgery or trauma in terms of pathomechanisms, immune responses, and repair mechanisms. Consequently, no single animal model can fully reflect the complexity of clinical cases.

Finally, clinical trial design and evaluation criteria present further challenges in the translational process. While preclinical studies primarily rely on liver function biomarkers, pathological assessments, and laboratory results to evaluate therapeutic efficacy, clinical trials incorporate more complex evaluation metrics, such as long-term patient survival, complication rates, and quality of life. Additionally, assessing the sustainability of treatment effects and designing individualized treatment regimens are particularly challenging in clinical trials.

In summary, the translation of therapeutic approaches for hepatic ischemia-reperfusion injury from preclinical models to clinical trials faces several significant hurdles, including model discrepancies, challenges with dosage and timing, preclinical diversity, and the complexity of clinical trial design. To overcome these challenges, future research must prioritize interdisciplinary collaboration, optimize the selection of animal models, and incorporate more individualized and dynamic assessment methods in clinical trials.

The complexity of hepatic IRI demands a paradigm shift from linear pathway inhibition to systems-level modulation of cell death networks. By targeting convergent nodes of DAMP release while addressing metabolic comorbidities such as steatosis, next-generation therapies may transform reperfusion injury from an unavoidable paradox into a clinically manageable phenomenon. Realizing this vision will require large-scale multicenter trials integrating multi-omics profiling and advanced organ preservation technologies, ultimately paving the way for precision medicine in liver transplantation and hepatobiliary surgery.

## LIST OF ABBREVIATIONS

IRI: Ischemia-Reperfusion Injury
DAMPs: Damage-Associated Molecular Patterns
ROS: Reactive Oxygen Species
cGAS: Cyclic GMP-AMP Synthase
cGAMP: Cyclic GMP-AMP
STING: Stimulator Interferon Genes
TXNIP: Thioredoxin-Interacting Protein
IR: Ischemia Reperfusion
Dvl2: Dishevelled-2
Me1: Malic Enzyme 1
NAC: N-acetylcysteine
NMP: Normothermic Machine Pperfusion
HOPE: Hypothermic Oxygenated Perfusion
